# Automated procedure for slice thickness verification of computed tomography images: Variations of slice thickness, position from iso‐center, and reconstruction filter

**DOI:** 10.1002/acm2.13317

**Published:** 2021-06-09

**Authors:** Nani Lasiyah, Choirul Anam, Eko Hidayanto, Geoff Dougherty

**Affiliations:** ^1^ Department of Physics Faculty of Sciences and Mathematics Diponegoro University Semarang Indonesia; ^2^ Department of Applied Physics and Medical Imaging California State University Channel Islands Camarillo CA USA

**Keywords:** AAPM CT performance phantom, CT scanner, CT scanner performance evaluation, slice sensitivity profile, slice thickness

## Abstract

**Purpose:**

The purpose of this study is to automate the slice thickness verification on the AAPM CT performance phantom and validate it for variations of slice thickness, position from iso‐center, and reconstruction filter.

**Methods:**

An automatic procedure for slice thickness verification on AAPM CT performance phantom was developed using MATLAB R2015b. The stair object image within the phantom was segmented, and the middle stair object was located. Its angle was determined using the Hough transformation, and the image was rotated accordingly. The profile through this object was obtained, and its full‐width of half maximum (FWHM) was automatically measured. The FWHM indicated the slice thickness of the image. The automated procedure was applied with variations in three independent parameters, i.e., the slice thickness, the distance from the phantom to the iso‐center, and the reconstruction filter. The automated results were compared to manual measurements made using electronic calipers.

**Results:**

The differences of the automated results from the nominal slice thicknesses were within 1.0 mm. The automated results are comparable to those from manual approach (i.e., the difference of both is within 12%). The automatic procedure accurately obtained slice thickness even when the phantom was moved from the iso‐center position by up to 4 cm above and 4 cm below the iso‐center. The automated results were similar (to within 0.1 mm) for various reconstruction filters.

**Conclusions:**

We successfully developed an automated procedure of slice thickness verification and confirmed that the automated procedure provided accurate results. It provided an easy and effective method of determining slice thickness.

## 1 INTRODUCTION

Computed tomography (CT) has the ability to precisely locate organs^1^, ^2^ and detect abnormalities, such as tumors, within the body.^3^ It is a non‐invasive, quick, and painless technique with good spatial resolutions (both in‐plane [*x‐y*] and cross‐plane [*z*])^4^, ^5^ and good contrast.^6^ CT imaging depends on many parameters, from pre‐image processing until post‐image processing, to ensure image quality. The parameters used in pre‐image processing define the resulting image quality, which then affect the decision on treatment.^7^, ^8^, ^9^ These parameters include the reconstruction field of view (FOV), the effective mAs, the reconstruction algorithm, beam collimation, and slice thickness.^9^, ^10^

Slice thickness is one of the important parameters, and it has to be optimized as needed. Slice thickness affects the cross‐plane resolution of the clinical image, which then impacts the accuracy of the size determination of the organ.^11^, ^12^ The slice thickness also directly impacts image noise. Decreasing the reconstructed slice thickness increases image noise.^13^ To compensate for increased noise, the operator may choose to increase the mAs (dose) to the patient.^14^

The accuracy of slice thickness determination has been investigated in previous studies using various phantoms.^15^, ^16^, ^17^, ^18^, ^19^, ^20^ In the AAPM CT performance phantom, slice thickness is measured as the thickness of a stair object using electronic calipers.^21^, ^22^, ^23^ A more objective measurement of slice thickness can be achieved by determining the full‐width at half maximum (FWHM) of the pixel profile across the stair objects.^24^ However, this manual approach is tedious and time‐consuming. An automated procedure would increase the measurement speed and objectivity. An automated procedure for slice thickness verification on the AAPM CT performance system utilizing MATLAB software was previously proposed by Sofiyatun et al.^21^ They showed that automated procedure can produce a more accurate estimate than manually calculated results.^21^ However, the study was only conducted for one slice thickness value, i.e., 5 mm. In this paper, we validated automated slice thickness results for various slice thicknesses, phantom positions from the iso‐center, and reconstruction filters.

## 2 MATERIALS AND METHODS

### 2.1 CT scanner and phantom

**Fig. 1:**
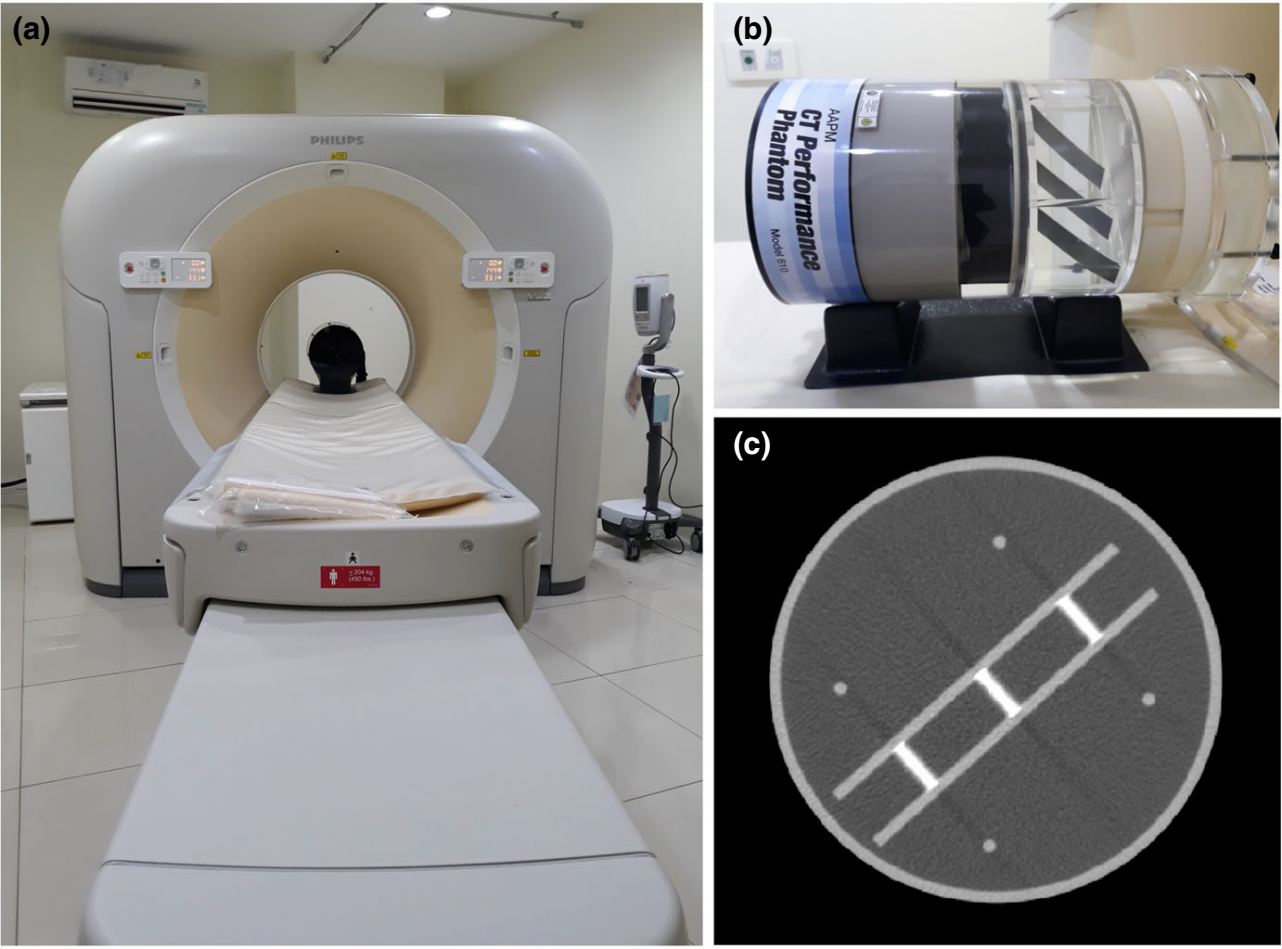
(a) Philips 128‐slice CT scanner (b) AAPM CT performance phantom, and (c) a CT image of the phantom.

The study was conducted at the Radiological Installation of the Diponegoro National Hospital (RSND), using a Philips Ingenuity 128‐slice CT scanner [Fig. 1(a)] and the AAPM CT performance phantom (Model 610, CIRS, Virginia, US) [Fig. 1(b)]. The objects for slice thickness measurement were aluminum plates each of size 0.635 mm × 25.4 mm, surrounded by water. Figure 1(c) shows an axial image of the phantom. The AAPM CT performance phantom was scanned with three different variables: nominal slice thickness, position from the iso‐center, and reconstruction filter. The respective acquisition parameters are listed in Table 1.

**Table 1:** Acquisition parameters for the variations of slice thickness, position from iso‐center, and reconstruction filter.

Acquisition parameter	Variation of slice thickness	Variation of position from iso‐center	Variation of reconstruction filter
Tube potential	120 kV	120 kV	120 kV
Tube current	200 mA	200 mA	200 mA
Mode	Helical	Helical	Helical
Pitch	0.984	0.984	0.984
Field of view (FOV)	260 mm	260 mm	260 mm
Rotation time	1 s	1 s	1 s
Filter reconstruction	Mid‐sternum	Mid‐sternum	Mid‐sternum, soft tissue, bone, cardiac, and brain
Position	At iso‐center	At iso‐center, 2 and 4 cm above, and 2 and 4 cm below	At iso‐center
Slice thickness	1, 2, 3, 4, and 5 mm	5 mm	5 mm

### 2.2 Automated measurement

**Fig. 2:**
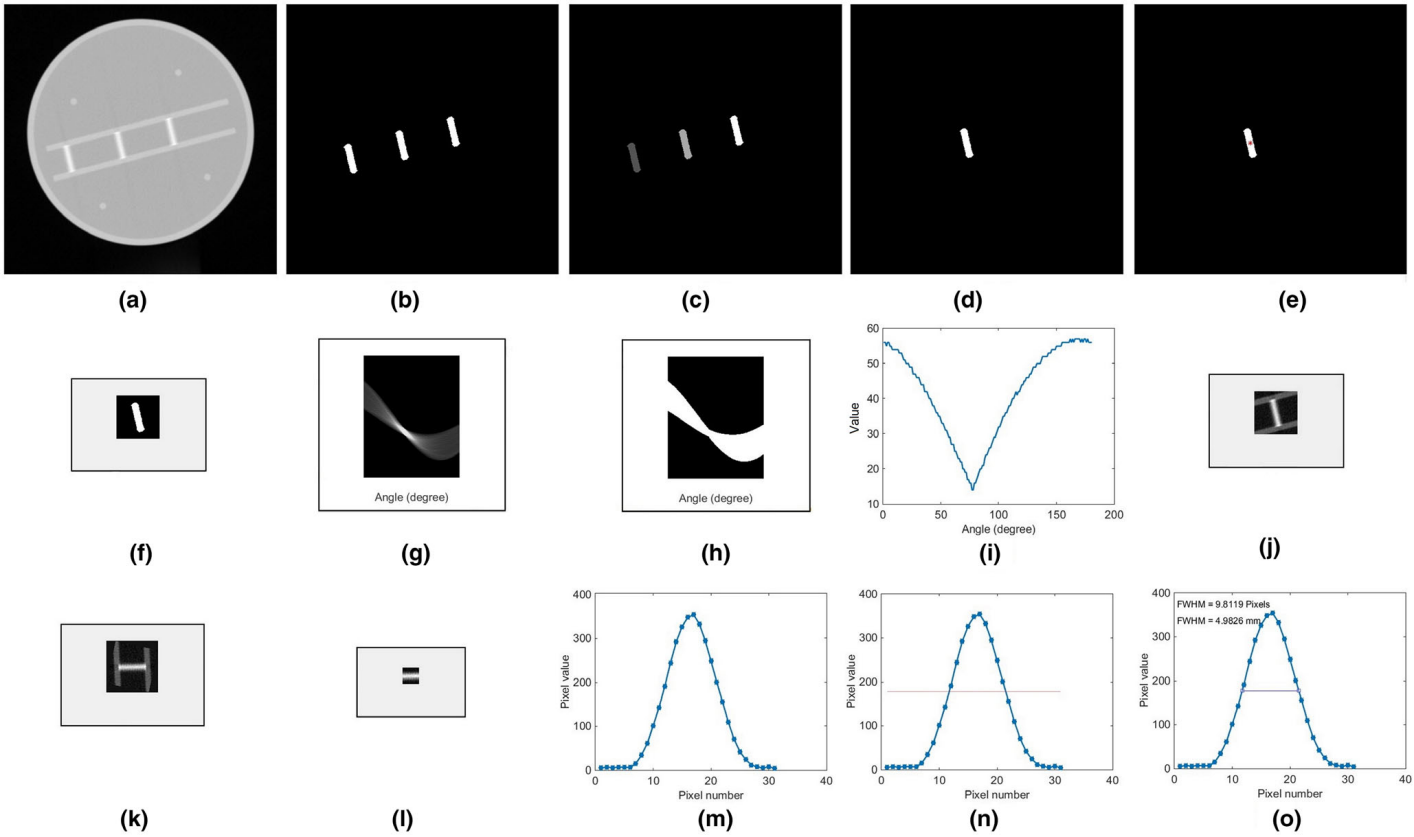
The steps of automated approach of slice thickness measurement: (a) CT image depicting the stairs object for determining the slice thickness, (b) segmentation of the stairs, (c) labeling the stairs, (d) the second object chosen (the middle object) #2, (e) red dot in the center position of the labeled object, (f) cropped image, (g) Hough transformation of cropped image, (h) normalized result of Hough transformation, (i) integration of Hough transformation to 1‐D to determine automatically the minimum angle, (j) cropped original image of the middle object #2, (k) image rotated by the minimum angle determined, (l) image re‐cropped to 30 × 30 pixels to eliminate the stair foundation, (m) average profile, (n) determining half‐maximum of the profile, and (o) the FWHM of the profile, which provides the slice thickness of the image.

The automated procedure for slice thickness verification was carried out utilizing a program developed in MATLAB R2015b. Figure 2 shows the program workflow for processing each image to obtain the FWHM value of the slice thickness.^21^

After segmenting, cropping and rotating the objects by an angle determined by the Hough Transformation, the profile across the objects [Fig. 2(l)] was created by averaging the pixel values in the *x*‐direction Fig. 2(m)]. The maximum profile value and the half‐maximum value were obtained [Fig. 2(n)], and the FWHM was calculated for each image in pixels [Fig. 2(o)], and converted to mm using the DICOM header conversion factor. All these steps were performed automatically by tapping a single button.^21^ The measurements for every variation were conducted on five frames, and the averages and standard deviations were calculated.

### 2.3 Manual measurement

**Fig. 3:**
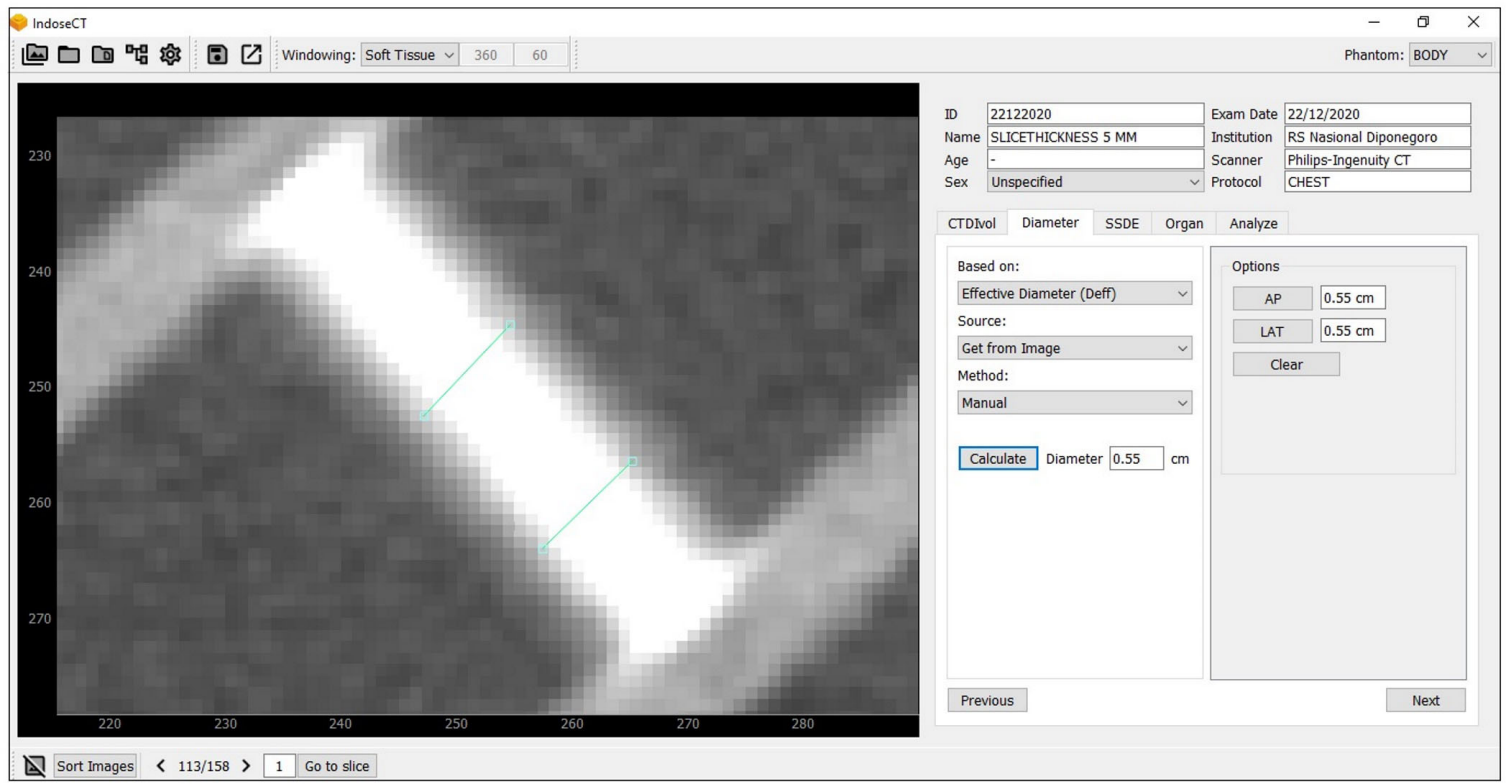
Screenshot of IndoseCT for manual slice thickness measurement.

The automated results of slice thicknesses were compared to manual measurements. Manual measurement of the slice thickness was carried out on IndoseCT software.^25^ Manual calculation was performed by measuring the thickness of the middle stair object using an electronic caliper. Both ends of the line drawn by the electronic caliper were placed on both sides of the middle stair object, and their boundaries were determined based on visual observation by the authors. Measurements were performed in three positions on the middle stair object after the image was zoomed‐in. Figure 3 shows the manual measurement based on our zoomed‐in view.

## 3 RESULTS

### 3.1 Slice thickness variation

**Fig. 4:**
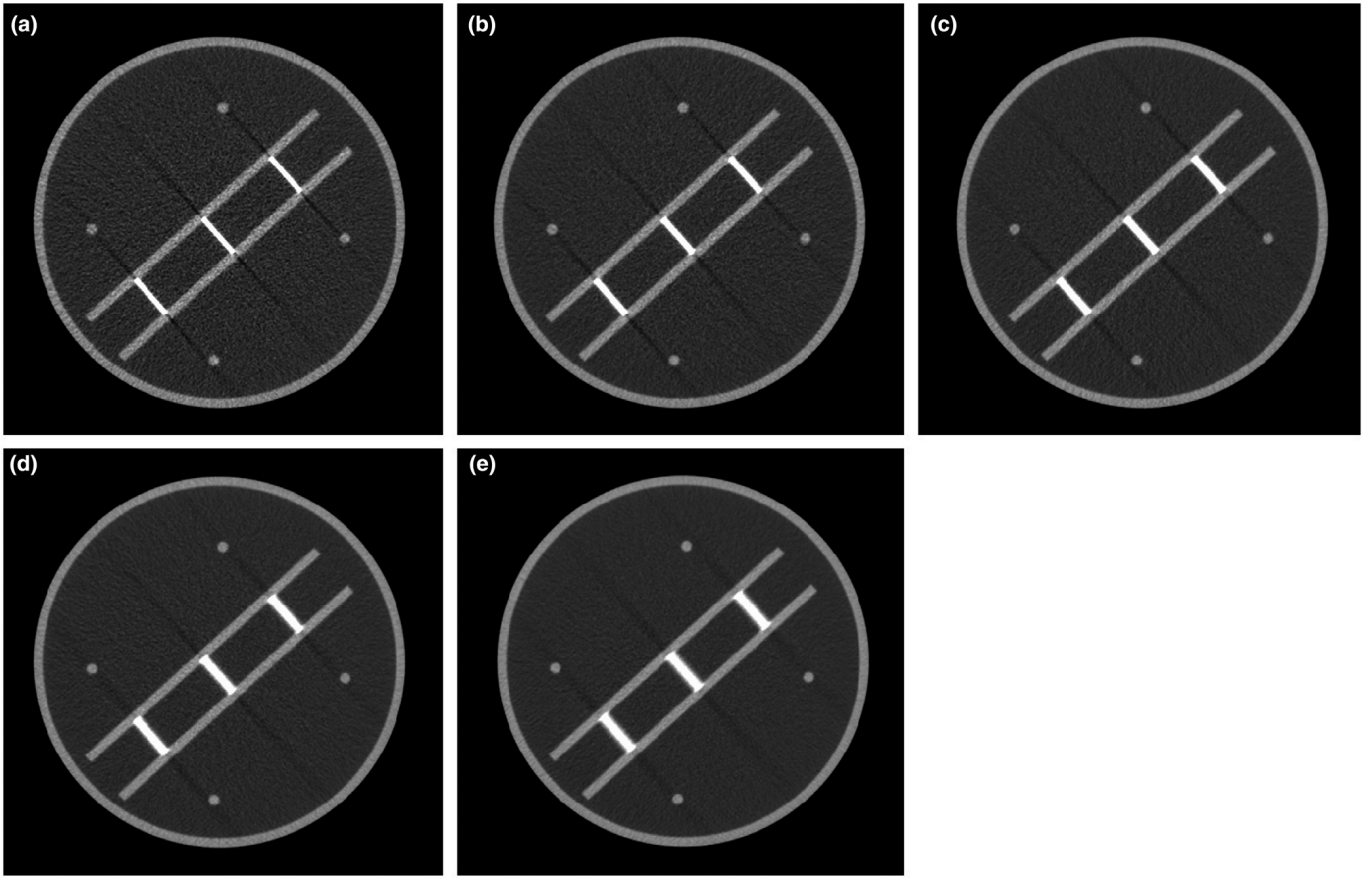
Images of the phantom for various slice thicknesses: (a) 1 mm, (b) 2 mm, (c) 3 mm, (d) 4 mm, and (e) 5 mm.

**Fig. 5:**
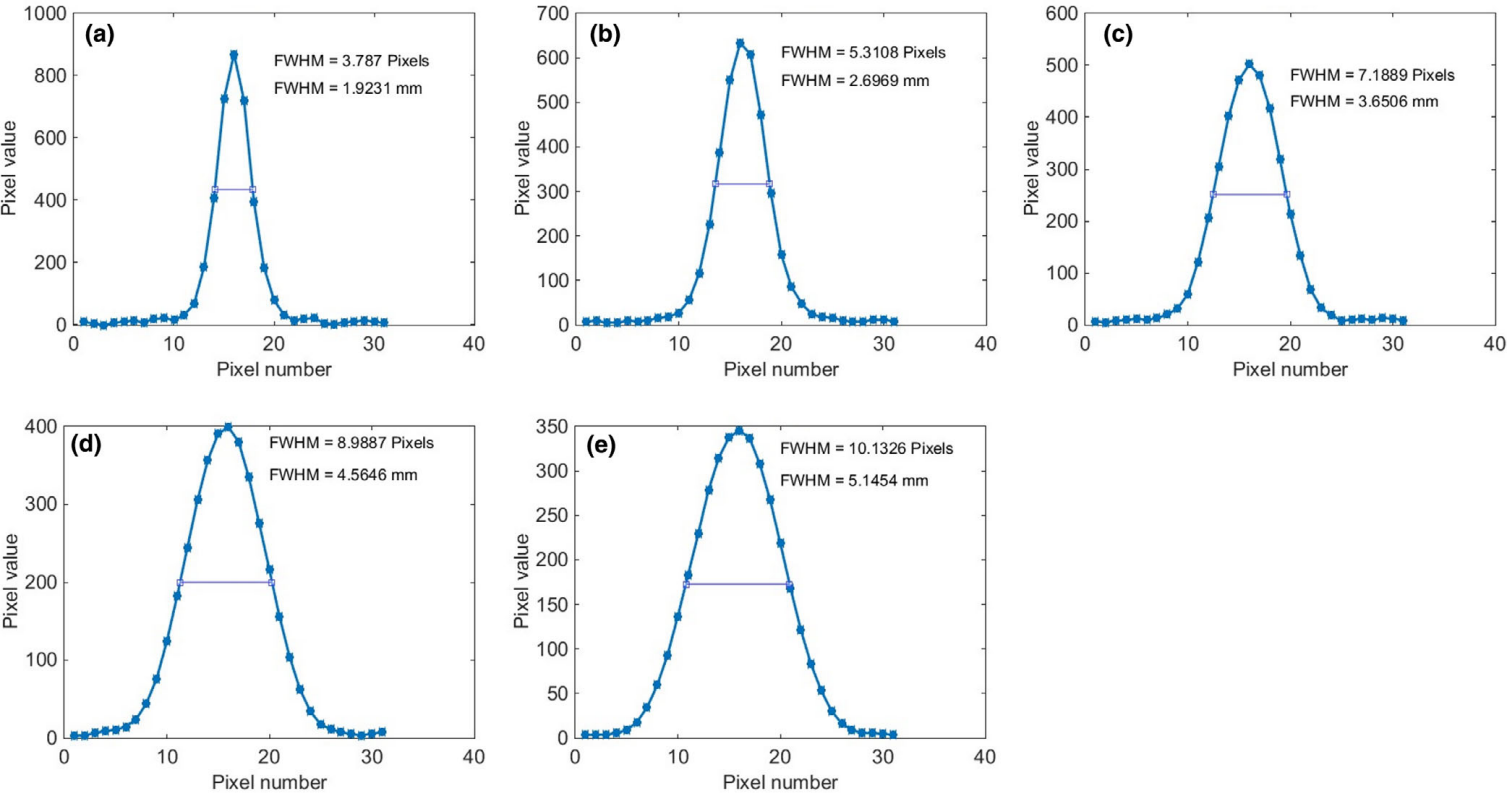
Profiles of the stair objects with FWHM values for various nominal slice thicknesses: (a) 1 mm, (b) 2 mm, (c) 3 mm, (d) 4 mm, and (e) 5 mm.

Figure 4 shows the image of the phantom for various nominal slice thicknesses from 1 to 5 mm. The corresponding profiles across the stair objects are shown in Fig. 5, along with the FWHM results for each slice thickness in units of both pixels and mm. The average values and standard deviations of both automated and manual calculations for various nominal slice thicknesses are tabulated in Table 2. The differences between the automated and manual results are less than 12%. The differences between the automated results and the nominal slice thicknesses are within 1.0 mm.

**Table 2:** Results of automated and manual calculations of the slice thickness for various slice thicknesses.

Set slice thickness	Slice thickness (mm)		Difference (%)
	Automated calculation	Manual calculation	
1 mm	2.0 ± 0.1	1.9 ± 0.1	5.0
2 mm	2.7 ± 0.0	2.7 ± 0.0	0.0
3 mm	3.5 ± 0.0	3.6 ± 0.2	2.9
4 mm	4.5 ± 0.1	4.7 ± 0.1	4.4
5 mm	5.1 ± 0.1	5.7 ± 0.3	11.8

### 3.2 Position from iso‐center variation

**Fig. 6:**
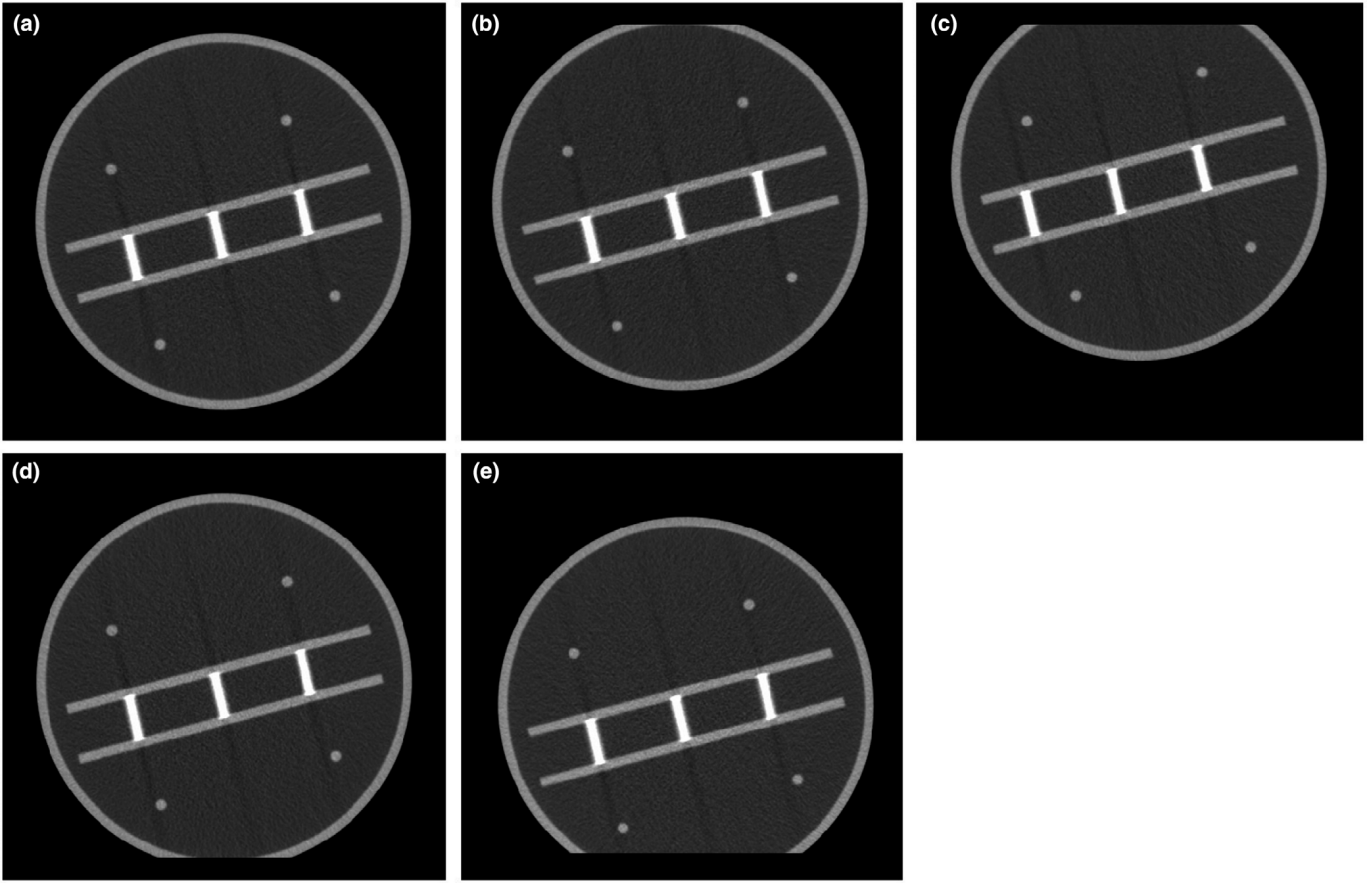
Images of the phantom for different phantom positions from the iso‐center: (a) at iso‐center, (b) 2 cm above the iso‐center, (c) 4 cm above the iso‐center, (d) 2 cm below the iso‐center, and (e) 4 cm below the iso‐center.

**Fig. 7:**
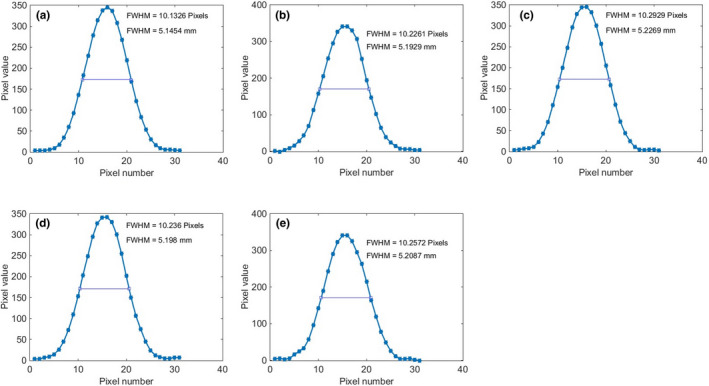
Stair object's pixels profiles and FWHM values for different phantom positions to the iso‐center: (a) center of iso‐center, (b) 2 cm above the iso‐center, (c) 4 cm above the iso‐center, (d) 2 cm below the iso‐center, and (e) 4 cm below the iso‐center.

Figure 6 shows the image of the phantom for various phantom positions from the iso‐center, and the corresponding profiles across the stair objects are shown in Fig. 7. The average values and standard deviations of both automated and manual calculations are tabulated in Table 3. The automatic method was able to accurately measure the slice thickness for various phantom positions from the iso‐center. For all positions, the differences of the automated results of slice thickness from the nominal slice thickness are less than 0.3 mm. The automated results are closer than the manual results to the nominal slice thickness.

**Table 3:** Results of automated and manual calculations of the slice thickness for various positions from the iso‐center.

Phantom position to iso‐center	Slice thickness (mm)		Difference (%)
	Automated calculation	Manual calculation	
Center	5.1 ± 0.1	5.7 ± 0.3	11.8
2 cm above	5.1 ± 0.1	5.7 ± 0.1	11.8
4 cm above	5.3 ± 0.3	5.7 ± 0.2	7.5
2 cm below	5.1 ± 0.2	5.7 ± 0.1	11.8
4 cm below	5.2 ± 0.1	5.6 ± 0.3	7.7

### 3.3 Reconstruction filter variation

**Fig. 8:**
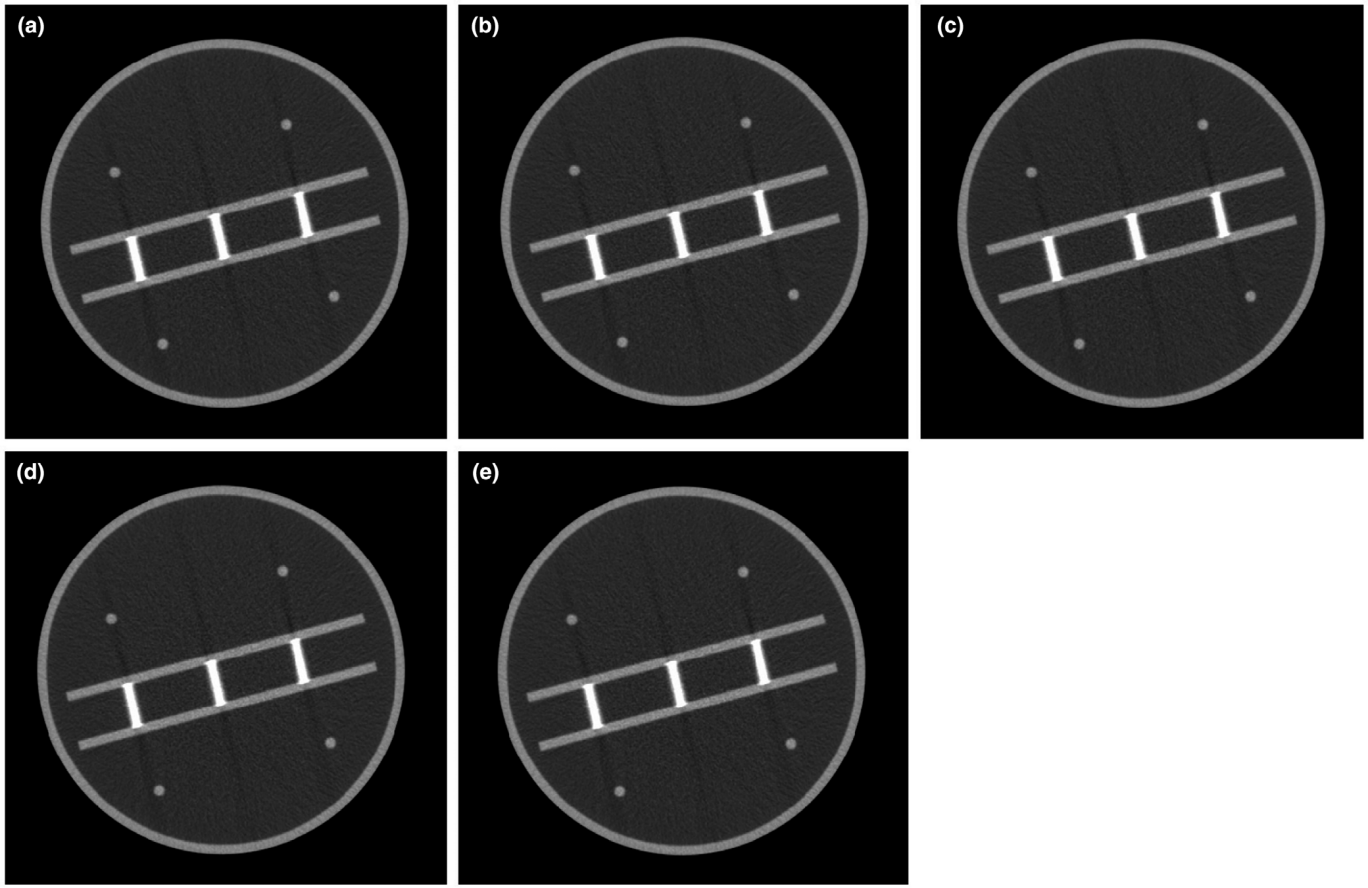
Images of the phantom in the center of iso‐center for various reconstruction filters: (a) mid‐sternum filter, (b) soft tissue reconstruction filter, (c) bone reconstruction filter, (d) cardiac reconstruction filter, and (e) brain reconstruction filter.

**Fig. 9:**
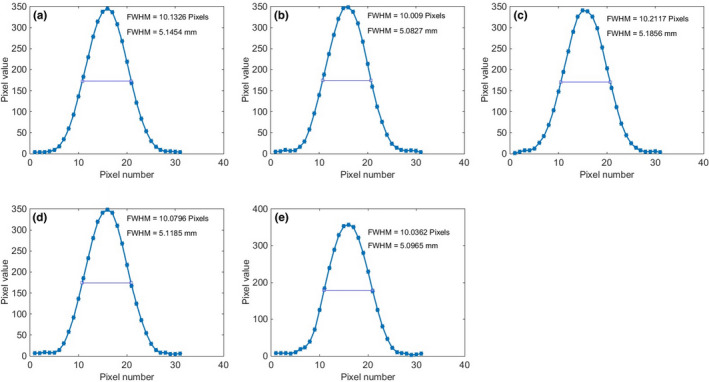
Stair object's profiles and FWHM values for various reconstruction filters: (a) lung reconstruction filter, (b) soft tissue reconstruction filter, (c) bone reconstruction filter, (d) cardiac reconstruction filter, and (e) brain reconstruction filter.

Figure 8 shows the image of the phantom for various image reconstruction filters, and the profiles across the stair objects are shown in Fig. 9. The average values and standard deviations for both automated and manual calculations for various image reconstruction filters are tabulated in Table 4. The automatic method is able to accurately measure the slice thickness for various image reconstruction filters. For all filters, the differences between the automated results and the nominal slice thicknesses are less than 0.1 mm.

**Table 4:** Results of automated and manual calculations of the slice thickness for various reconstruction filters.

Reconstruction filter	Slice thickness		Difference (%)
	Automated calculation	Manual calculation	
Mid‐sternum	5.1 ± 0.1	5.7 ± 0.3	11.8
Cardiac	5.1 ± 0.1	5.6 ± 0.2	9.8
Soft tissue	5.1 ± 0.1	5.4 ± 0.2	5.9
Bone	5.1 ± 0.1	5.7 ± 0.0	11.8
Brain	5.1 ± 0.1	5.7 ± 0.1	11.8

## 4 DISCUSSION

This study aims to develop and validate an automated procedure for the slice thickness verification from an AAPM CT performance phantom so that easier and more effective pre‐treatment measurement can be made. The automated procedure for slice thickness verification uses the thickness of the stair objects in an axial image.^21^ Increase thickness of the stairs results in a wider slice thickness.^22^

Previously, an automated slice thickness determination was proposed and implemented on one nominal slice thickness of 5 mm.^21^ This current study further validated the algorithm for several variations, i.e., slice thickness, position from iso‐center, and image reconstruction filter. We found that all nominal slice thicknesses from 1 to 5 mm, the differences between the automated results, and the nominal slice thicknesses are within the tolerance limit, i.e., 1.5 mm.^17^, ^26^

The current study confirmed that the results from the automated procedure are independent of position of the phantom from iso‐center. This suggests that users do not need to precisely locate the phantom in order to measure the slice thickness from the resulting image. This not only helps speed up image acquisition but also simplifies the image acquisition process. Another finding of the current study is that automated slice thickness results are not affected by the reconstruction filter used. This is different from the manual approach where the results may be affected by the reconstruction filter used, because user subjectivity in locating the border of the stair object may depend on the reconstruction filter.

The differences between the automated slice thickness results and manual results were within 12%. We found that the automated results were accurate, i.e., differences of less than 1 mm between them and the nominal slice thicknesses. Our automated method will be helpful in conducting a more convenient slice thickness verification. However, we need to validate its accuracy for different FOVs in a further study.

In this study, we focused on the middle stair object, assuming that its slice thickness value is no different from other two stair objects. The automated results of slice thickness may be affected by noise level, mode of acquisition (i.e., step and shot or helical modes), and pitch factor. All these parameters need to be investigated in future studies.

Apart from the slice thickness, the AAPM CT performance phantom has modules for measuring other CT performance parameters, such as noise, linearity of CT number, beam hardening, spatial in‐plane resolution, low contrast, and so on.^27^, ^28^ Developing an automated system for these parameters would greatly assist medical physicists in carrying out routine quality control.

## 5 CONCLUSIONS

We have proposed and validated the algorithm for an automated procedure for slice thickness verification on an AAPM CT performance phantom. We validated it for variations of slice thickness, positions from iso‐center, and reconstruction filter. The automated results are accurate, differing from the nominal thickness by less than 1.0 mm for slice thicknesses from 1 to 5 mm, for various positions, and for various reconstruction filters.
